# Dietary antioxidant capacity of the patients with cardiovascular disease in a cross-sectional study

**DOI:** 10.1186/s12937-015-0005-4

**Published:** 2015-03-15

**Authors:** Małgorzata E Zujko, Anna M Witkowska, Anna Waśkiewicz, Walerian Piotrowski, Katarzyna M Terlikowska

**Affiliations:** 1 Department of Food Commodities Science and Technology, Medical University, Szpitalna 37, Bialystok, 15-295 Poland; 2 Department of Epidemiology, Cardiovascular Disease Prevention and Health Promotion, Institute of Cardiology, Niemodlińska 33, Warsaw, 04-635 Poland

**Keywords:** Cardiovascular disease, Diet, Antioxidant capacity, Polyphenols, Flavonoids

## Abstract

**Background:**

The purpose of this study was to establish sources and patterns of antioxidant, polyphenol and flavonoid intakes in men and women with cardiovascular disease (CVD).

**Methods:**

The subjects with CVD and healthy controls (HC) were participants of the Polish National Multicenter Health Survey (WOBASZ). Food intakes were measured with the 1-day 24-hour recall method. A self-developed database was used to calculate dietary total antioxidant capacity (DTAC), dietary total polyphenol content (DTPC) and dietary total flavonoid content (DTFC).

**Results:**

DTAC did not differ between the men with CVD and HC men (6442 vs. 6066 μmol trolox equivalents – TE), but in the women with CVD it was significantly higher than in the HC women (6182 vs. 5500 μmol TE). The main sources of antioxidants in the males with CVD were: tea, coffee, apples, and nuts and seeds, and tea, coffee and apples in HC. In the females they were: tea, coffee, apples and strawberries, both in the women with CVD and HC. DTPC in the men with CVD did not differ from HC (1198 vs. 1114 mg gallic acid equivalents, GAE). In the females, DTPC was significantly higher in the subjects with CVD as compared to HC (1075 vs. 981 mg GAE). Predominant sources of polyphenols were: tea, coffee, cabbage, potatoes, apples and white bread in the men with CVD, and tea, coffee, potatoes, white bread and apples in HC, while in the women (both with CVD and HC): tea, coffee, apples, potatoes and cabbage. No differences in DTFC have been found between the males with CVD and HC (212 vs. 202 mg quercetine equivalents, QE). In the women with CVD, DTFC was significantly higher than in HC (200 vs. 177 mg QE). Main sources of flavonoids in all participants (men and women, CVD and HC) were tea, apples, cabbage and coffee.

**Conclusions:**

Polish men and women faced with CVD beneficially modify their dietary practices by enhancing intakes of foods that are sources of antioxidants, polyphenols and flavonoids. Different sources and patterns of antioxidant, polyphenol and flavonoid intakes, however, between male and female patients with CVD were observed.

## Background

Cardiovascular disease (CVD) is one of the most common causes of death worldwide, with pathogenesis in which multiple fixed (age, gender, genotype, menopausal status) and modifiable factors (diet, exercise, stress, smoking and ethanol consumption) are involved [1]. Among the modifiable lifestyle-related factors the main role plays a diet [2]. Epidemiological studies found an inverse association between the intake of antioxidant-rich food and the risk of CVD [3,4]. Dietary antioxidants such as vitamin E, vitamin C, carotenoids and polyphenols have protective cardiovascular effect through suppressing oxidative stress, defined as an imbalance between production of reactive oxygen (ROS) and nitrogen species (RNS), and antioxidants (endo- and exogenous). Adverse effects of free radical (FR) overproduction on cardiovascular system are associated with endothelial dysfunction mediated through nitric oxide (NO) degradation, lipid peroxidation and inflammatory response. Antioxidants can donate electrons to FR molecules, that contain one or more unpaired electrons, blocking by this deleterious chain reactions [5].

Polyphenols which are derived mostly from plant foods, are widely distributed in the human diet. Dietary total polyphenol intake could be as high as 1 g/d, which is much higher than all other classes of phytochemicals and known dietary antioxidants e.g. antioxidant vitamins [6]. The major class of polyphenols are flavonoids. Several *in vitro* and *in vivo* studies have suggested that dietary flavonoids, which are mainly found in fruits and vegetables, may induct an antioxidant defense system and exert beneficial effects on the vascular system via an improving endothelial function and inhibiting low density lipoprotein oxidation [7,8].

Adequate nutrition, which supplies appropriate ratios of antioxidants, plays important role in the CVD prevention and can improve symptoms of the disease. Therefore, the purpose of this study was to estimate dietary total antioxidant capacity (DTAC), dietary total polyphenol (DTPC) and flavonoid contents (DTFC) in the diet of the CVD patients, and to establish main dietary patterns of antioxidants. To our knowledge this is the first attempt to estimate antioxidants in a cross-sectional Polish study, which involves a representative sample of CVD patients.

## Methods

### Subjects and food consumption

Subjects were participants of the Polish National Multicenter Health Survey (WOBASZ), which was carried out by the National Institute of Cardiology in Warsaw, Poland, in cooperation with five Polish medical universities. The WOBASZ study was approved by the Bioethics Committee of the National Institute of Cardiology in Warsaw (no 708). This study was continued for 3 years, from January 2003 to December 2005, and included a representative random sample of the general Polish population. The rationale, design, and methods of the cross-sectional study have been described in detail elsewhere [9-11].

A sample of 19,200 individuals (men and women) from the general population of more than 26 million Polish inhabitants aged 20–74 years was randomly selected. Two small (up to 8 thousand inhabitants), 2 medium (8–40 thousand inhabitants), and 2 large communes (above 40 thousands inhabitants) from each of the sixteen Polish provinces were randomly selected. One hundred men and 100 women from each commune, were randomly chosen from the personal identification number (PESEL) database. Finally, 13545 people (6392 men and 7153 women) agreed to take part in the study. Of these, approx. 50% was randomly selected for nutrition study. Lastly, from 6661 adults (3132 males and 3529 females) 1-day 24-hour dietary recalls (1-d 24-h DR) were collected. The interviewers were trained by a qualified nutritionist specialized in performing nutrition surveys.

**Figure 1:**
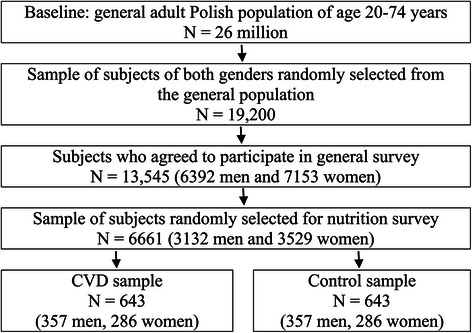
**Scheme of the study and subjects’ selection procedure.**

Approx. 10% of the studied sample, 643 subjects (357 men and 286 women), have been previously diagnosed by a cardiologist and hospitalized for CVD (heart attack, heart failure, stroke, heart defects, cardiac arrhythmia, coronary angioplasty or coronary artery bypass, heart pacemaker). This information was provided from self-reported health status questionnaires. Subjects could report more than one disease. A control group with no diagnosed CVD was randomly chosen from 6661 adults by a propensity score matching (PSM) technique, based on matching characteristics: age, body mass index (BMI), cigarette smoking, physical activity, commune type, marital status, level of education, household *per-capita* income, self-rated health. Scheme of the study and subjects’ selection procedure are presented in Figure 1. Baseline characteristics of the participants are shown in Table 1.

**Table 1:** **Baseline characteristics of the participants**

Characteristics	Men			Women		
	CVD patients	Healthy control	p	CVD patients	Healthy control	p
Number	357	357	-	286	286	-
Number of CVD types^a^						
Heart attack	170	-	-	82	-	-
Heart failure	138	-	-	116	-	-
Stroke	44	-	-	22	-	-
Heart defects	103	-	-	94	-	-
Cardiac arrhythmia	169	-	-	171	-	-
Coronary angioplasty or bypass	43	-	-	7	-	-
Heart pacemaker	7	-	-	3	-	-
Age (years)	57.2 ± 11.8	57.0 ± 11.8	0.82	57.5 ± 12.0	57.1 ± 12.4	0.70
BMI (kg/m^2^)	27.8 ± 4.9	27.8 ± 4.7	0.92	29.1 ± 5.9	28.8 ± 6.3	0.56
Hypertension^b^ (%)	64.7	56.6	0.03	64.0	54.2	0.02
Hypercholesterolemia^c^ (%)	71.5	74.6	0.36	72.1	77.0	0.19
Total cholesterol (mmol/l)	5.44 ± 1.15	5.61 ± 1.14	0.05	5.54 ± 1.14	5.72 ± 1.13	0.06
HDL (mmol/l)	1.26 ± 0.36	1.35 ± 0.42	0.01	1.42 ± 0.37	1.50 ± 0.40	0.02
Triglycerides (mmol/l)	1.68 ± 1.04	1.76 ± 1.31	0.42	1.67 ± 1.24	1.61 ± 1.66	0.66
Diabetes^d^ (%)	16.4	12.0	0.09	16.6	11.1	0.06
Smoking (%)			0.02			0.15
*Non-smokers*	24.4	30.5		65.0	62.9	
*Former smokers*	47.6	37.0		22.0	18.5	
*Current smokers*	28.0	32.5		12.9	18.5	
Physical activity (%)			0.94			0.09
*Low level*	44.5	45.1		59.4	55.9	
*Middle level*	12.0	11.2		6.6	11.9	
*High level*	43.4	43.7		33.9	32.2	
Commune type (%)			0.78			0.59
*<8 thousand inhabitants*	30.5	31.7		32.5	33.2	
*8-40 thousand inhabitants*	37.3	34.7		37.1	33.2	
*>40 thousand inhabitants*	32.2	33.6		30.4	33.6	
Marital status (%)			0.02			0.22
*Married*	86.3	79.8		67.8	62.9	
*Single*	13.7	20.2		32.2	37.1	
Level of education (%)			0.74			0.16
*Under middle*	66.1	66.4		62.9	65.4	
*Middle*	25.8	24.1		29.0	30.4	
*Higher*	8.1	9.5		8.0	4.2	
Household per-capita income (%)			0.03			0.82
*Low*	36.7	42.9		47.5	46.1	
*Middle*	42.3	31.6		38.5	39.9	
*High*	11.2	13.7		6.3	7.7	
*Not stated*	9.8	11.8		7.7	6.3	
Self-rated health (%)			0.69			0.63
*Bad, average*	65.8	64.4		74.5	76.2	
*Good, very good*	34.2	35.6		25.5	23.8	

^a^Patients could indicate more than one disease, ^b^Hypertension: systolic blood pressure ≥ 140 mmHg or diastolic blood pressure ≥ 90 mmHg or treatment in interview, ^c^Hypercholesterolemia: total cholesterol ≥ 5 mmol/l or treatment in interview, ^d^Diabetes: fasting glucose ≥ 7 mmol/l or treatment in interview, BMI – Body Mass Index, CVD – cardiovascular disease, HDL – high-density lipoprotein.

The survey was conducted individually in men and women due to gender differences in burden of CVD, which include prevalence of hypertension, diabetes mellitus, metabolic syndrome and lifestyle factors [12].

Each participant was informed about the screening procedures and signed the agreement form. A standardized questionnaire was designed for data collection, which was suitable for the objectives of the study. The respondents were questioned about social, demographic, and economic status, physical activity, smoking, cardiovascular disease, diabetes, health awareness, health care, any ongoing medical treatments. Additionally, the height and weight of each patient were measured and the body mass index (BMI) was calculated according to the formula: BMI = weight(kg)/height(m)^2^. Blood pressure measurements were taken three times on the right arm after 5 minutes of rest in a sitting position in one minute intervals and a final value was calculated as an average measurement. Biochemical tests: total cholesterol, high-density lipoprotein (HDL), triglycerides and fasting glucose, were performed at the Central Laboratory of the National Institute of Cardiology in Warsaw.

### Procedure of food extraction

Food products were randomly purchased in triplicate at different Polish food markets. Edible raw parts of foods were dried and pulverized in a grinder. Samples (0.25 g) were placed in test tubes with 10 ml of methanol/water solution (50:50, v/v) and the pH was adjusted to 2 using 2 M HCl. The tubes were thoroughly shaken at room temperature for 1 hour and then centrifuged at 4000 *g* for 10 min in a centrifuge (MPW-350e, Poland). The resulting supernatants were collected in clean dry test tubes. Then the residues were extracted with 10 ml of an acetone/water mixture (70/30, v/v) and the procedure was repeated. Both methanol and acetone extracts were combined and used for analyses. Samples of tea, coffee, drinking chocolate and hot cocoa were extracted with water (1 g sample per 100 ml boiling distilled water).

### Procedure of FRAP assay

Ferric Reducing Antioxidant Power (FRAP) was determined according to Benzie and Strain [13]. A 1.5 ml of 2,4,6-tripyridyl-s-triazine solution was warmed to 37°C; then a reagent blank was measured at 593 nm. Subsequently, 50 μl of the sample, dissolved in distilled water (1:4), was added to the FRAP reagent. The absorbance was measured following incubation at 37°C for 4 min. The antioxidant potential of samples was determined from the standard curve and expressed as trolox equivalents (mmol TE/100 g fresh edible mass of plant foods or 100 ml of beverage).

### Procedure of total polyphenol assay

Total polyphenol contents in food products were determined using a Singleton and Rossi method [14]. Samples (0.2 ml) were mixed with a 1 ml Folin-Ciocalteau reagent previously diluted in distilled water (1:10) and 0.8 ml of 7.5% (w/v) sodium carbonate. The absorbance was measured after 30 min at 765 nm. The results were expressed as gallic acid equivalents (GAE) in mg total polyphenols on 100 g fresh edible mass of plant foods or 100 ml of beverages.

### Procedure of total flavonoid assay

Total flavonoid content in food samples was determined according to Arvouet-Grand et al. [15]. Briefly, 1 ml of 2% aluminium trichloride (AlCl_3_) in methanol was mixed with the same volume of the extract. A yellow color indicated the presence of flavonoids. Absorption readings at 415 nm were taken after 10 min against a blank sample consisting of a 1 ml extract solution with 1 ml methanol without AlCl_3_. The concentration of total flavonoids in samples was determined from the standard curve and expressed as quercetin equivalents (mg QE/100 g fresh mass of plant foods or 100 ml of beverage).

### Assessment of total polyphenols and flavonoids intake, and antioxidant capacity of diet

Previously published [16-19] self-developed dietary database of the total antioxidant capacity of foods, determined by the FRAP method, as well as total polyphenols and flavonoids in plant food items was used to calculate dietary total antioxidant capacity (DTAC), dietary total polyphenol content (DTPC) and dietary total flavonoid content (DTFC). This database contains over 150 national foods and food products classified into 6 categories (1 – beverages; 2 – vegetables; 3 - fruits and jams; 4 - bread, rolls and cereals products; 5 – chocolates; 6 - nuts and seeds) of which 84 plant foods were found to be consumed by the participants. DTAC was expressed as μmol trolox equivalents (TE), DTPC – as mg gallic acid equivalents (GAE) and DTFC – as mg quercetine equivalents (QE).

### Statistical analysis

Statistical analyses were performed using SAS software (version 9.2; Cary, NC, USA). The results were expressed as number, percentage, mean value and standard deviation or 95% confidence interval, median and 25-75% percentiles. Categorical variables were compared with the chi-square test. Normality of continuous data distribution was verified with the Shapiro-Wilk test. To compare the quantitative variables with normal distribution the *t* test was used, and for nonnormal distribution the Mann–Whitney-Wilcoxon nonparametric test was applied. P-values of less than 0.05 were considered statistically significant.

## Results

Baseline characteristics of the participants are shown in Table 1. Compared to the healthy control (HC), hypertension was significantly frequent, both in men and women with CVD. Among biochemical parameters serum HDL levels were found lower in CVD patients (both men and women) in comparison to HC. Among lifestyle variables, significant influences were observed for smoking, marital status and household per-capita income in the CVD and HC men.

Daily intakes of plant foods and beverages by the participants are shown in Table 2. Among beverages, consumption of tea and coffee was predominant, with 67-69% and 20-28% contribution, respectively, in all studied groups (Table 3). The men presented 29% higher consumption of vegetables, but 40% lower fruit and fruit jam intake in comparison to the women. Moreover, the women with CVD consumed significantly higher amounts of fruit and fruit jams than the HC women. Potato intake accounted for 53-62% consumption of vegetables, while apples for 58-66% of fruit eaten in all groups. Cereals and cereal products consumption was 47% higher in the men than in the women, with respective 56-63% and 45-49% contributions of white bread. Minor intakes of chocolates, nuts and seeds were observed in all studied subjects, although the men with CVD were 2-fold more frequent consumers of these foods.

**Table 2:** **Plant food and beverage intakes in the study groups (g [ml] fresh edible mass/person/day)**

Plant food and beverages	Men				P	Women				P
	CVD patients (n = 357)		Healthy control (n = 357)			CVD patients (n = 286)		Healthy control (n = 286)		
	Mean 95% CI	Median 25-75 percentile	Mean 95% CI	Median 25-75 percentile		Mean 95% CI	Median 25-75 percentile	Mean 95% CI	Median 25-75 percentile	
**Beverages** ^1^	584.3 (546–622)	500 (400–750)	627.6 (589–666)	600 (400–800)	0.09	528.8 (496–562)	500 (350–750)	509.0 (478–540)	500 (250–700)	0.38
tea infusion^2^	403.8 (373–435)		421.6 (392–451)			363.8 (335–393)		341.1 (316–366)		
coffee infusion^3^	119.7 (102–137)		124.4 (108–141)			138.7 (120–158)		141.1 (122–161)		
others^4^	60.8 (41–80)		81.5 (58–105)			26.3 (15–38)		26.9 (17–37)		
**Vegetables**	521.2 (486–557)	515 (266–728)	560.5 (526–594)	527 (329–729)	0.12	415.9 (383–449)	386 (187–602)	421.9 (387–457)	386 (190–598)	0.93
potatoes	307.7 (280–336)		346.1 (318–375)			218.6 (194–244)		246.9 (220–274)		
cabbage^5^	50.3 (41–60)		46.2 (37–56)			41.0 (33–49)		37.9 (31–45)		
tomatoes	43.1 (34–52)		42.0 (34–50)			49.4 (39–60)		33.8 (25–42)		
carrots	24.0 (20–28)		26.9 (22–32)			24.1 (18–30)		23.8 (19–29)		
onions	15.5 (13–18)		15.2 (13–17)			11.8 (10–14)		11.9 (10–14)		
beetroots	11.8 (8–16)		13.4 (9–18)			10.0 (6–14)		8.9 (5–13)		
cauliflower	4.6 (1–8)		4.6 (2–7)			8.3 (3–13)		3.3 (1–6)		
others^6^	64.2 (56–72)		66.9 (59–73)			52.6 (46–59)		55.3 (47–63)		
**Fruit and jams**	194.3 (171–217)	140 (0–300)	167.4 (146–189)	104 (0–255)	0.06	238.3 (208–269)	178 (15–328)	181.3 (158–205)	150 (5–280)	0.02
apples	129.3 (111–147)		100.6 (84–117)			138.2 (116–160)		106.1 (91–121)		
bananas	7.1 (3–11)		12.2 (7–18)			11.8 (7–17)		10.9 (7–15)		
strawberries	10.6 (5–17)		7.5 (3–12)			20.8 (7–35)		13.8 (5–23)		
plums	8.5 (4–13)		13.6 (8–19)			11.3 (7–16)		11.7 (4–20)		
pears	7.9 (3–12)		6.6 (2–11)			9.1 (3–15)		3.0 (0–6)		
citrus fruits^7^	8.5 (3–14)		7.4 (3–11)			12.0 (5–19)		7.6 (2–13)		
grapes	5.6 (1–10)		3.1 (0.4-6)			12.0 (5–19)		9.2 (4–14)		
sour cherries	4.6 (2–7)		1.2 (0.3-2)			5.1 (1–9)		5.2 (2–8)		
others^8^	12.1 (9–16)		15.1 (7–23)			18.0 (11–25)		13.9 (9–18)		
**Cereal products**	198.8 (187–211)	184 (128–253)	201.9 (188–216)	183 (115–261)	0.98	138.4 (129–148)	122 (81–180)	136.5 (126–147)	126 (72–179)	0.84
white bread	112.2 (101–124)		128.0 (115–141)			61.6 (53–70)		66.8 (58–76)		
others^9^	86.6 (77–96)		73.9 (63–84)			76.8 (68–86)		69.6 (62–78)		
**Chocolates** ^10^	1.6 (0.6-2.7)	0	1.2 (0.4-2)	0	0.69	0.7 (0.2-1.2)	0	0.6 (0.1-1.1)	0	0.62
**Nuts and seeds** ^11^	1.7 (0–3.5)	0	0.9 (0.1-1.7)	0	0.99	0.7 (0–1.5)	0	0.6 (0–1.4)	0	0.07

n – number; CVD – cardiovascular disease; CI – confidence interval; ^1^tea infusion, coffee infusion, drinking chocolate, hot cocoa −1 g sample per 100 ml boiling distilled water; ^2^tea infusion: black tea, green tea, red tea, rooibos tea, white tea; ^3^coffee infusion: instant coffee, ground coffee; ^4^others: beer, apple juice, orange juice, red wine, white wine, black currant juice, lemon juice, hot cocoa, drinking chocolate; ^5^cabbage: white, red cabbage and Chinese cabbage; ^6^others: cucumber, parsley roots, leeks, celery roots, fennel, sorrel, chives, red and green pepper, lettuce, radish, tomato paste, ketchup, green beans, beans, peas, button mushrooms; ^7^citrus fruits: oranges, grapefruits, mandarins; ^8^others: sweet cherries, apricots, red currants, nectarines, peaches, kiwi fruits, watermelon, northern cranberries, bilberries, woodland strawberries, lingonberries, raspberries, jams (orange, bilberry, plum, black currant, sour cherry, strawberry, apricot, peach, pineapple); ^9^others: wheat rolls, wheat four, wholegrain bread, noodles, buckwheat groats, barley groats, extruded rye bread, extruded graham bread, oats, rice; ^10^chocolates: dark, semisweet, milk, white; ^11^nuts and seeds: walnuts, sunflower seeds, pistachios, hazelnuts, peanuts, pumpkin seeds.

**Table 3:** **Contribution of individual plant foods and beverages to intake of respective categories (%)**

Plant food and beverages	Men		Women	
	CVD patients	Healthy control	CVD patients	Healthy control
**Beverages** ^**1**^	100	100	100	100
tea infusion^2^	69	67	69	67
coffee infusion^3^	20	20	27	28
others^4^	11	13	4	5
**Vegetables**	100	100	100	100
potatoes	59	62	53	59
cabbage^5^	10	9	10	9
tomatoes	9	8	12	8
carrots	5	5	6	6
onions	3	3	3	3
beetroots	3	3	3	3
cauliflower	1	1	2	1
others^6^	13	12	13	14
**Fruit and jams**	100	100	100	100
apples	66	60	58	58
bananas	4	7	5	6
strawberries	5	4	9	8
plums	4	8	5	6
pears	4	4	4	2
citrus fruits^7^	5	4	5	4
grapes	3	2	5	5
sour cherries	2	1	2	3
others^8^	7	10	7	8
**Cereal products**	100	100	100	100
white bread	56	63	45	49
others^9^	44	37	55	51

CVD – cardiovascular disease.

For ^1–9^, see Table 2.

Dietary total antioxidant capacity in the studied groups is shown in Table 4. Mean DTAC in the men with CVD was 6442 μmol TE/person/day and 6066 μmol TE/person/day in the HC men, with the highest contribution of tea (34%), coffee (14%), apples (9%), and nuts and seeds (9%) in the subjects with CVD and tea (38%), coffee (16%) and apples (7%) in HC (Table 5). Despite lower DTAC in the HC men, antioxidant capacity of consumed beverages was significantly higher in HC vs. the men with CVD. Mean DTAC in the women with CVD was significantly higher than in the HC women (6182 vs. 5500 μmol TE/person/day, respectively) and it was associated with significantly higher antioxidant capacity of consumed fruit and fruit jams in the women with CVD. The highest intakes of antioxidants in the women with CVD and the HC women were: tea (32 vs. 34%), coffee (17 vs. 20%), apples (10 vs. 8%), and strawberries (7 vs. 6%).

**Table 4:** **Dietary total antioxidant capacity in the study groups (**μ**mol TE/person/day)**

Plant food and beverages	Men				P	Women				P
	CVD patients (n = 357)		Healthy control (n = 357)			CVD patients (n = 286)		Healthy control (n = 286)		
	Mean 95% CI	Median 25-75 percentile	Mean 95% CI	Median 25-75 percentile		Mean 95% CI	Median 25-75 percentile	Mean 95% CI	Median 25-75 percentile	
**Beverages** ^1^	3287 (3080–3493)	3050 (2035–4290)	3537 (3335–3739)	3300 (2200–4683)	0.04	3213 (3009–3417)	3059(2200–4296)	3081 (2888–3274)	2753 (1933–4125)	0.31
tea infusion^2^	2221(2049–2393)		2319(2156–2482)			2001(1841–2161)		1876(1736–2016)		
coffee infusion^3^	925.0 (788–1062)		961.8 (837–1087)			1072 (926–1218)		1091 (940–1241)		
others^4^	140.6 (97–184)		256.5 (163–350)			140.2 (70–210)		114.9 (66–164)		
**Vegetables**	730.5 (655–806)	562 (321–888)	729.2 (660–798)	578 (336–858)	0.79	614.3 (544–685)	478 (265–719)	562.9 (501–625)	452 (244–741)	0.41
potatoes	184.6 (1680201)		207.6 (191–225)			131.2 (116–146)		148.2 (132–164)		
cabbage^5^	161.0 (111–212)		117.7 (85–151)			112.6 (74–151)		106.1 (71–141)		
tomatoes	64.7 (51–78)		63.0 (51–76)			74.1 (58–90)		50.7 (38–63)		
carrots	36.0 (30–42)		40.4 (33–47)			36.2 (27–45)		35.8 (28–43)		
onions	35.7 (29–42)		35.0 (30–40)			27.2 (23–32)		27.4 (23–32)		
beetroots	120.4 (79–162)		137.1 (87–187)			102.3 (57–147)		90.6 (51–131)		
cauliflower	24.7 (6–43)		25.0 (10–40)			44.8 (17–72)		18.0 (5–31)		
others^6^	103.4 (86–120)		103.3 (87–120)			85.9 (70–101)		86.1 (70–102)		
**Fruit and jams**	1374(1177–1571)	726 (0–1980)	1105(937–1272)	644 (0–1540)	0.05	1865(1490–2240)	1100 (64–2300)	1445(1166–1724)	660 (43–1926)	0.03
apples	568.7 (489–649)		442.7 (371–514)			608.2 (512–704)		466.7 (399–534)		
bananas	24.3 (11–37)		41.6 (23–60)			40.1 (22–58)		37.1 (22–52)		
strawberries	235.4 (103–367)		166.2 (61–271)			460.1 (157–763)		306.0 (110–502)		
plums	95.5 (47–144)		152.9 (93–213)			126.1 (75–177)		130.8 (39–222)		
pears	18.9 (8–29)		15.8 (5–27)			21.9 (8–36)		7.1 (0–14)		
citrus fruits^7^	77.7 (28–127)		62.1 (26–98)			103.6 (39–168)		61.2 (18–104)		
grapes	60.8 (13–109)		33.6 (4–63)			129.9 (52–207)		99.3 (43–155)		
sour cherries	152.5 (82–223)		40.1 (11–69)			167.1 (45–289)		172.3 (71–274)		
others^8^	140.3 (80–200)		149.8 (70–230)			207.8 (112–304)		164.3 (72–256)		
**Cereal products**	353.5 (328–379)	306 (204–432)	354.8 (327–383)	316 (192–448)	0.89	260.1 (233–287)	197 (126–310)	246.7 (221–273)	200 (124–303)	0.74
white bread	190.8 (171–210)		217.6 (196–239)			104.8 (91–119)		113.6 (99–129)		
others^9^	162.7 (139–187)		137.1 (111–163)			155.3 (128–182)		133.1 (109–157)		
**Chocolates** ^10^	136.1 (42–230)	0	78.5 (27–130)	0	0.69	65.4 (15–115)	0	48.1 (9–87)	0	0.62
**Nuts and seeds** ^11^	561.2 (0–1306)	0	262.1 (29–495)	0	0.99	164.7 (0–350)	0	116.6 (0–281)	0	0.74
**Total**	6442(5624–7260)	5676(3926–7156)	6066(5688–6445)	5356(4013–7199)	0.65	6182(5714–6651)	5643(3887–7271)	5500(5102–5898)	4754(3619–6606)	0.01

n – number, CI – confidence interval, CVD – cardiovascular disease, TE – Trolox equivalents.

For ^1–11^, see Table 2.

**Table 5:** **Percentage contribution of the respective categories and individual plant food and beverage items to DTAC, DTPC and DTFC (%)**

Plant food and beverages	DTAC (% contribution)				DTPC (% contribution)				DTFC (% contribution)			
	Men		Women		Men		Women		Men		Women	
	CVD patients	Healthy control	CVD patients	Healthy control	CVD patients	Healthy control	CVD patients	Healthy control	CVD patients	Healthy control	CVD patients	Healthy control
**Beverages** ^1^	**51**	**58**	**52**	**56**	**35**	**41**	**38**	**40**	**39**	**43**	**40**	**42**
tea infusion^2^	34	38	32	34	24	27	24	25	29	32	28	29
coffee infusion^3^	14	16	17	20	9	10	12	13	8	9	10	12
others^4^	2	4	2	2	2	3	2	2	1	2	2	1
**Vegetables**	**12**	**12**	**10**	**10**	**26**	**25**	**22**	**23**	**30**	**28**	**25**	**26**
potatoes	3	3	2	3	8	10	6	8	6	7	4	5
cabbage^5^	3	2	2	2	9	5	6	6	14	10	10	10
tomatoes	1	1	1	1	2	2	3	2	3	2	3	2
carrots	<1	<1	<1	<1	<1	<1	<1	<1	1	1	1	1
onions	<1	<1	<1	<1	1	1	1	1	3	3	2	3
beetroots	2	2	2	2	2	2	2	2	1	2	1	1
cauliflower	<1	<1	<1	<1	<1	<1	<1	<1	<1	<1	<1	<1
others^6^	2	2	1	2	3	4	3	3	3	3	2	3
**Fruit and jams**	**21**	**18**	**30**	**27**	**19**	**17**	**27**	**23**	**25**	**21**	**31**	**27**
apples	9	7	10	8	8	7	10	8	18	14	20	17
bananas	<1	<1	<1	<1	<1	1	1	1	<1	<1	<1	<1
strawberries	4	3	7	6	2	2	5	3	2	1	3	2
plums	1	3	2	2	1	2	2	2	1	2	2	2
pears	<1	<1	<1	<1	<1	<1	<1	<1	<1	<1	<1	<1
citrus fruits^7^	1	1	2	1	1	1	2	1	1	1	2	1
grapes	1	<1	2	2	<1	<1	2	2	<1	<1	1	1
sour cherries	2	<1	3	3	2	<1	2	3	<1	<1	1	1
others^8^	2	2	3	3	2	2	3	2	<1	1	1	1
**Cereal products**	**5**	**6**	**4**	**4**	**14**	**14**	**11**	**12**	**6**	**6**	**4**	**5**
white bread	3	4	2	2	6	8	4	5	3	4	2	2
others^9^	2	2	2	2	7	6	7	7	2	2	2	2
**Chocolates** ^10^	**2**	**1**	**1**	**<1**	**2**	**1**	**1**	**<1**	**<1**	**<1**	**<1**	**<1**
**Nuts and seeds** ^11^	**9**	**5**	**3**	**2**	**4**	**2**	**1**	**1**	**<1**	**<1**	**<1**	**<1**
**Total**	**100**	**100**	**100**	**100**	**100**	**100**	**100**	**100**	**100**	**100**	**100**	**100**

CVD – cardiovascular disease, DTAC – dietary total antioxidant capacity, DTPC – dietary total polyphenol content, DTFC – dietary total flavonoid content.

For ^1–11^, see Table 2.

DTPC in the study groups is presented in Table 6. DTPC in the males with CVD was 1198 mg GAE/person/day vs. 1114 mg GAE/person/day in HC, with the highest contribution of tea (24%), coffee (9%), cabbage (9), potatoes (8%), apples (8%) and white bread (6%) in CVD patients, and tea (27%), coffee (10%), potatoes (10%), white bread (8%) and apples (7%) in the HC subjects (Table 5). DTPC in the females was significantly higher in the subjects with CVD in comparison to HC (1075 vs. 981 mg GAE/person/day, respectively), and was associated with higher consumption of polyphenols from fruit and fruit jams. Predominant sources of polyphenols in the women with CVD and in the HC women were: tea (24 vs. 25%), coffee (12 vs. 13%), apples (10 vs. 8%), potatoes (6 vs. 8%) and cabbage (6 vs. 6%).

**Table 6:** **Dietary total polyphenol intake in the study groups (mg GAE/person/day)**

Plant food and beverages	Men				P	Women				P
	CVD patients (n = 357)		Healthy control (n = 357)			CVD patients (n = 286)		Healthy control (n = 286)		
	Mean 95% CI	Median 25-75 percentile	Mean 95% CI	Median 25-75 percentile		Mean 95% CI	Median 25-75 percentile	Mean 95% CI	Median 25-75 percentile	
**Beverages** ^1^	421.1 (395–447)	388 (279–541)	451.9 (426–477)	426 (284–594)	0.05	405.1 (380–431)	378 (279–541)	389.2 (365–413)	355 (232–533)	0.35
tea infusion^2^	286.7 (265–309)		299.3 (278–320)			258.3 (238–279)		242.2 (224–260)		
coffee infusion^3^	111.3 (95–128)		115.7 (101–131)			129.0 (111–147)		131.2 (113–149)		
others^4^	23.1 (16–30)		36.9 (25–48)			17.8 (10–26)		15.8 (10–22)		
**Vegetables**	322.1 (255–389)	228 (136–338)	280.8 (240–321)	230 (151–338)	0.63	236.7 (184–289)	186 (100–255)	228.8 (182–276)	179 (96–283)	0.65
potatoes	95.4 (87–104)		107.3 (98–116)			67.8 (60–76)		76.6 (68–85)		
cabbage^5^	109.7 (45–175)		54.3 (17–92)			60.7 (11–110)		59.2 (15–104)		
tomatoes	25.9 (21–31)		25.2 (20–30)			29.7 (23–36)		20.3 (15–25)		
carrots	8.4 (7–10)		9.4 (8–11)			8.4 (6–11)		8.3 (7–10)		
onions	16.9 (14–20)		16.6 (14–19)			12.9 (11–15)		13.1 (11–15)		
beetroots	21.1 (14–28)		24.1 (15–33)			18.0 (10–26)		15.9 (9–23)		
cauliflower	4.1 (1–7)		4.2 (2–7)			7.5 (3–12)		3.1 (1–5)		
others^6^	40.4 (35–46)		39.8 (35–45)			31.8 (27–37)		32.5 (28–37)		
**Fruit and jams**	224.5 (196–253)	136 (0–348)	187.6 (162–214)	115 (0–267)	0.06	288.6 (241–337)	193 (11–385)	227.8 (189–266)	125 (5–299)	0.02
apples	99.6 (85–114)		77.5 (65–90)			106.4 (90–123)		81.7 (70–94)		
bananas	7.4 (3–11)		12.7 (7–18)			12.3 (7–18)		11.3 (7–16)		
strawberries	25.5 (11–40)		18.0 (7–29)			49.7 (17–83)		33.1 (12–54)		
plums	16.9 (8–25)		27.1 (16–38)			22.3 (13–31)		23.1 (7–39)		
pears	5.7 (3–9)		4.7 (2–8)			6.6 (2–11)		2.1 (0–4)		
citrus fruits^7^	12.7 (5–21)		10.5 (5–16)			17.3 (7–28)		10.4 (3–18)		
grapes	9.6 (2–17)		5.3 (1–10)			20.4 (8–33)		15.6 (7–24)		
sour cherries	23.4 (13–34)		6.2 (2–11)			25.7 (7–44)		26.5 (11–42)		
others^8^	23.8 (16–32)		25.7 (13–39)			27.8 (17–39)		24.1 (13–35)		
**Cereal products**	161.9 (152–172)	146 (100–203)	159.1 (148–170)	142 (91–209)	0.39	119.3 (110–129)	100 (64–152)	115.2 (106–125)	102 (64–147)	0.69
white bread	76.3 (69–84)		87.1 (78–96)			41.9 (36–48)		45.4 (39–51)		
others^9^	85.6 (76–95)		72.1 (62–82)			77.4 (68–87)		69.7 (61–79)		
**Chocolates** ^10^	21.5 (7–36)	0	12.6 (4–21)	0	0.69	10.2 (2–18)	0	7.7 (1–14)	0	0.62
**Nuts and seeds** ^11^	46.6 (0–107)	0	22.4 (3–41)	0	0.99	15.1 (0–31)	0	11.9 (0–27)	0	0.74
**Total**	1198 (1096–1299)	1034 (775–1356)	1114 (1051–1177)	999 (768–1312)	0.46	1075 (997–1152)	956 (703–1254)	981 (909–1052)	865 (644–1117)	0.01

n – number, CI – confidence interval, CVD – cardiovascular disease, GAE - gallic acid equivalents.

For ^1–11^, see Table 2.

DTFC in the men with CVD was 212 mg QE/person/day vs. 202 mg QE/person/day in HC (Table 7). The males with CVD presented significantly higher consumption of flavonoids from beverages and lower intake of flavonoids from fruit and fruit jams than HC. The main sources of flavonoids both in the men with CVD and in HC were: tea (29 vs. 32%), apples (18 vs. 14%), cabbage (14 vs. 10%), coffee (8 vs. 9%) and potatoes (6 vs.7%) (Table 5). Dietary flavonoid intake in the women with CVD was significantly higher as compared to the HC women, and it was dependent on the higher consumption of flavonoids from fruit and fruit jams. The predominant flavonoid sources in the women with CVD vs. the HC women were: tea (28 vs. 29%), apples (20 vs. 17%), coffee (10 vs. 12%) and cabbage (10 vs. 10%).

**Table 7:** **Dietary total flavonoid intake in the study groups (mg QE/person/day)**

Plant food and beverages	Men				P	Women				P
	CVD patients (n = 357)		Healthy control (n = 357)			CVD patients (n = 286)		Healthy control (n = 286)		
	Mean 95% CI	Median 25-75 percentile	Mean 95% CI	Median 25-75 percentile		Mean 95% CI	Median 25-75 percentile	Mean 95% CI	Median 25-75 percentile	
**Beverages** ^1^	82.6 (77.4-87.7)	77 (56–114)	88.4 (83.5-93.3)	77 (60–115)	0.04	79.2 (74.3-84.1)	77 (53–107)	75.8 (71.2-80.3)	75 (41–103)	0.34
tea infusion^2^	61.8 (57.0-66.6)		64.5 (60.0-69.0)			55.7 (51.2-60.1)		52.2 (48.3-56.1)		
coffee infusion^3^	17.7 (15.1-20.3)		18.4 (16.0-20.8)			20.5 (17.7-23.3)		20.9 (18.0-23.8)		
others^4^	3.1 (2.1-4.0)		5.5 (3.5-7.4)			3.0 (1.6-4.5)		2.7 (1.7-3.7)		
**Vegetables**	63.8 (54.4-73.1)	40 (23–65)	57.1 (49.7-64.5)	39 (25–62)	0.98	49.4 (41.8-56.9)	31 (17–52)	45.3 (38.4-52.3)	29 (17–48)	0.56
potatoes	12.0 (10.9-13.1)		13.5 (12.4-14.6)			8.5 (7.5-9.5)		9.6 (8.6-10.7)		
cabbage^5^	28.8 (19.8-37.8)		20.1 (13.0-27.2)			19.9 (13.1-26.6)		17.1 (10.7-23.4)		
tomatoes	5.3 (4.2-6.4)		5.1 (4.1-6.1)			6.0 (4.8-7.3)		4.1 (3.1-5.1)		
carrots	2.1 (1.8-2.5)		2.4 (2.0-2.8)			2.1 (1.6-2.7)		2.1 (1.7-2.5)		
onions	6.5 (5.4-7.6)		6.4 (5.4-7.3)			4.9 (4.1-5.8)		5.0 (4.2-5.8)		
beetroots	3.0 (2.0-4.0)		3.4 (2.2-4.7)			2.5 (1.4-3.7)		2.3 (1.3-3.3)		
cauliflower	0.3 (0.1-0.6)		0.3 (0.1-0.5)			0.6 (0.2-0.9)		0.2 (0.1-0.4)		
others^6^	5.8 (5.1-6.5)		5.9 (5.2-6.7)			4.8 (4.1-5.5)		4.9 (4.2-5.7)		
**Fruit and jams**	52.5 (46.2-58.8)	37 (0–87)	43.2 (37.6-48.9)	30 (0–69)	0.03	62.6 (54.3-70.8)	43 (1–88)	47.6 (41.3-54.0)	41 (1–73)	0.02
apples	37.4 (32.1-42.6)		29.1 (24.4-33.8)			39.9 (33.6-46.3)		30.7 (26.2-35.1)		
bananas	0.9 (0.4-1.3)		1.5 (0.8-2.1)			1.4 (0.8-2.1)		1.3 (0.8-1.8)		
strawberries	3.2 (1.4-5.0)		2.3 (0.8-3.7)			6.2 (2.1-10.4)		4.2 (1.5-6.8)		
plums	2.6 (1.3-4.0)		4.2 (2.6-5.9)			3.5 (2.1-4.9)		3.6 (1.1-6.1)		
pears	1.2 (0.6-1.9)		1.0 (0.3-1.8)			1.4 (0.5-2.4)		0.5 (0–1.0)		
citrus fruits^7^	2.7 (1.0-4.4)		2.0 (0.8-3.2)			3.4 (1.2-5.6)		2.0 (0.5-3.4)		
grapes	1.1 (0.2-2.0)		0.6 (0.1-1.2)			2.4 (1.0-3.8)		1.8 (0.8-2.9)		
sour cherries	1.7 (0.9-2.5)		0.5 (0.1-0.8)			1.9 (0.5-3.3)		2.0 (0.8-3.1)		
others^8^	1.7 (1.1-2.2)		2.1 (0.9-3.4)			2.3 (1.4-3.2)		1.7 (1.0-2.4)		
**Cereal products**	12.0 (11.3-12.8)	11 (7–16)	12.3 (11.4-13.1)	11 (7–16)	0.89	8.4 (7.8-9.1)	7 (5–11)	8.1 (7.5-8.8)	7 (4–11)	0.71
white bread	7.0 (6.2-7.7)		7.9 (7.1-8.7)			3.8 (3.3-4.3)		4.1 (3.6-4.7)		
others^9^	5.1 (4.4-5.7)		4.3 (3.6-5.1)			4.6 (4.0-5.3)		4.0 (3.4-4.6)		
**Chocolates** ^10^	0.4 (0.1-0.7)	0	0.2 (0.1-0.4)	0	0.69	0.2 (0.04-0.3)	0	0.1 (0.03-0.3)	0	0.62
**Nuts and seeds** ^11^	0.6 (0–1.4)	0	0.3 (0.1-0.6)	0	0.99	0.2 (0.001-0.4)	0	0.2 (0–0.5)	0	0.74
**Total**	212 (199–225)	190 (131–260)	202 (191–212)	180 (133–239)	0.38	200 (187–213)	178 (124–243)	177 (167–187)	166 (116–220)	0.03

n – number, CI – confidence interval, CVD – cardiovascular disease, QE - quercetine equivalents.

For ^1–11^, see Table 2.

## Discussion

Diets that teem with antioxidants are relevant to body antioxidant status and are essential for prevention and alleviation of oxidative stress-related symptoms [20,21]. Polyphenols and flavonoids, which belong to this group of compounds, are plentiful dietary antioxidants, that may protect against oxidative damage, reducing by this risk of various diseases [22]. Despite this, a number of studies, that estimate flavonoid, polyphenol or antioxidant-rich food intakes is narrow, or the reports on their major dietary sources are limited to some populations.

Previous studies carried out in the US [23] and Spanish [24] populations used the most extensive USDA flavonoid database to estimate intakes of individual flavonoids or used phenol-explorer database [25] to approximate intakes of polyphenols other than the flavonoids. To calculate dietary total antioxidant capacity, some authors [26] has recently used a published USDA database for ORAC (Oxygen Radical Absorbance Capacity) in selected foods. Others [27] developed an antioxidant food database by using FRAP assay. The present study estimates both volume and patterns of dietary total antioxidant consumption together with total polyphenol and flavonoid intakes in the CVD patients. Phenolic contents in foods are influenced by genetic, environmental and processing factors, therefore use of dietary database that contains indigenous plant foods seemed to be more adequate. Therefore, this study uses a self-developed dietary database that contains total antioxidant capacity, polyphenol and flavonoid contents in plant foods [16-19]. For some beverages and plant foods, which were consumed by the participants, but were not included in this database (such as different types of tea), additional analyses were performed.

Flavonoid intakes in our survey (range 177–212 mg QE/day) are in agreement with some studies conducted in the US population – 190 mg/day [23], but lower compared to the Spanish population – 313 mg/day [24]. Our present findings concerning polyphenol intakes (range 981–1198 mg GAE/day) are comparable to our previous study performed in the general Polish population (range 1031–1172 mg GAE/day) [18], and also with other studies carried out in the French (1193 mg/day) [28], Finnish (863 mg/d) [29], and Spanish (1171 mg/d) [30] populations, but lower in comparison to some Polish study, which aimed to examine participants from the city of Krakow (1757 mg/day) [31]. For DTAC, results in the current study (range 5500–6442 μmol TE) are similar to a Spanish study (6014 μmol TE analyzed by the FRAP method) [30], but higher in comparison to our previous research conducted in diabetic patients (range 4271–5697 μmol TE by the FRAP method) [20].

The present study established that DTAC was significantly higher in the female CVD patients vs. HC, whereas no differences has been found in the men. The differences in the women can be explained by higher fruit intakes in the women with CVD, as well as by naturally high total antioxidant capacity of consumed fruits, which resulted from the total polyphenol and flavonoid contents.

The main contributors to the DTAC, DTPC and DTFC values in all studied groups were: tea, coffee and apples, as well as cabbage, strawberries and potatoes, because of high consumption of these foods in the Polish population [18,19]. Surprisingly, despite generally low nut and seed intake in the Polish population, these foods were one of the major contributors to DTAC in the men with CVD. On the contrary, dietary intake in the women with CVD was teeming with apples and strawberries. It is likely that CVD patients paid more attention to adequate nutrition due to CVD diagnosis. A number of studies showed that nuts and strawberries improved antioxidant status in humans, and their consumption was inversely associated with CVD risk and mortality [32-34]. As it has been demonstrated previously nuts and seeds (mainly walnuts) and strawberries were characterized by high antioxidant capacities as well as by high polyphenol contents [17]. Therefore, despite minor nut and seed intakes and moderate seasonal consumption of strawberries, these foods provide essential sources of antioxidants in a diet. Dietary intakes of antioxidants, however, are dependent on seasonal variability of consumption, *e.g.* intake of berry fruits such as strawberries and raspberries in Poles is the highest in the summer period [26]. This research, however, was conducted throughout the year, therefore it recorded virtually essential and representative sources of antioxidants for the studied population.

According to the literature, main contributors to the flavonoid, polyphenol and antioxidant intakes vary in different populations. Major flavonoid sources in the US diet [23], for example were: tea, citrus juices, wine and citrus fruits, whereas apples, red wine, unspecified fruits and oranges in the Spanish diet [24]. As regards to polyphenol intakes, nonalcoholic beverages and fruits were the most important contributors in the French population [28], while coffee and cereals in the Finnish [29], and coffee, oranges, apples, olives, olive oil and red wine by contrast in the Spanish population at a high cardiovascular risk [35]. As to sources of antioxidants, their major contributors in the Norwegian population [36] were: coffee, tea, red wine, blueberries, walnuts, oranges, cinnamon and broccoli, while in the US population [37] they were: tea, dietary supplements, fruits and fruit juices.

The authors are aware of some limitations of this research. At first, food intakes in this study have been estimated with the 1-day 24-hour dietary recall method, that does not reflect habitual or long-term food intakes. Twenty-four-hour recall, however, is a common method, which is useful to estimate mean food intakes in large groups of participants and by this it is suitable for contrasting the dietary status of a group with different levels of risk factors of certain diseases [38]. In this study, however, the number of participants was less than 1300, but taking into account the fact that the sample selection reflected the general adult Polish population, it seemed to be the most suitable for the objectives of this study. One of the limitations of this cross-sectional study is the fact that some patients may have been more likely to die and thus were excluded from this study thereby introducing bias. Subjects excluded in this manner could have had different dietary patterns or food preferences compared with subjects without cardiovascular disease. Finally, this study might have underestimated some polyphenol, flavonoid and antioxidant intakes from a few specific foods, such as spices for example, and it also did not analyze changes during the food preparation.

## Conclusions

This study demonstrates different sources and patterns of antioxidant, polyphenol and flavonoid intakes in male and female patients with CVD. The dietary intakes of antioxidants, polyphenols and flavonoids were higher in the males with CVD as compared to the healthy male individuals, but the differences were not significant. The men with CVD generally consumed more antioxidants, polyphenols and flavonoids from fruits and jams, chocolates, nuts and seeds, but less from beverages. In the women with CVD, however, significantly higher antioxidant, polyphenol and flavonoid intakes from all food categories surveyed were observed as compared to the healthy women. These results demonstrate attempts made by the studied representative samples of Polish men and women with CVD to introduce beneficial modifications to their dietary practices by enhancing intakes of foods that are sources of antioxidants, polyphenols and flavonoids.

## Abbreviations

1-d 24-h DR: 1-day 24-hour dietary recalls
BMI: Body mass index
CVD: Cardiovascular disease
DTAC: Dietary total antioxidant capacity
DTFC: Dietary total flavonoid content
DTPC: Dietary total polyphenol content
FRAP: Ferric reducing antioxidant power
GAE: Gallic acid equivalents
HC: Healthy controls
HDL: High-density lipoprotein
ORAC: Oxygen radical absorbance capacity
PSM: Propensity score matching technique
QE: Quercetine equivalents
TE: Trolox equivalents
